# Past, present, and future trends of overweight and obesity in Belgium using Bayesian age-period-cohort models

**DOI:** 10.1186/s12889-022-13685-w

**Published:** 2022-07-07

**Authors:** Robby De Pauw, Manu Claessens, Vanessa Gorasso, Sabine Drieskens, Christel Faes, Brecht Devleesschauwer

**Affiliations:** 1 Department of Epidemiology and Public Health, Sciensano, Rue Juliette Wytsmanstraat 14, BE-1050 Brussels, Belgium; 2 Department of Rehabilitation Sciences, Ghent University, Ghent, Belgium; 3 Department of Public Health and Primary Care, Ghent University, Ghent, Belgium; 4 Data Science Institute, the Interuniversity Institute for Biostatistics and statistical Bioinformatics, Hasselt University, Hasselt, Belgium; 5 Department of Translational Physiology, Infectiology and Public Health, Ghent University, Merelbeke, Belgium

**Keywords:** Public health, Epidemiology, Projections, Obesity epidemic, Integrated nested Laplace approximation (INLA)

## Abstract

**Background:**

Overweight and obesity are one of the most significant risk factors of the twenty-first century related to an increased risk in the occurrence of non-communicable diseases and associated increased healthcare costs. To estimate the future impact of overweight, the current study aimed to project the prevalence of overweight and obesity to the year 2030 in Belgium using a Bayesian age-period-cohort (APC) model, supporting policy planning.

**Methods:**

Height and weight of 58,369 adults aged 18+ years, collected in six consecutive cross-sectional health interview surveys between 1997 and 2018, were evaluated. Criteria used for overweight and obesity were defined as body mass index (BMI) ≥ 25, and BMI ≥ 30. Past trends and projections were estimated with a Bayesian hierarchical APC model.

**Results:**

The prevalence of overweight and obesity has increased between 1997 and 2018 in both men and women, whereby the highest prevalence was observed in the middle-aged group. It is likely that a further increase in the prevalence of obesity will be seen by 2030 with a probability of 84.1% for an increase in cases among men and 56.0% for an increase in cases among women. For overweight, it is likely to see an increase in cases in women (57.4%), while a steady state in cases among men is likely. A prevalence of 52.3% [21.2%; 83.2%] for overweight, and 27.6% [9.9%; 57.4%] for obesity will likely be achieved in 2030 among men. Among women, a prevalence of 49,1% [7,3%; 90,9%] for overweight, and 17,2% [2,5%; 61,8%] for obesity is most likely.

**Conclusions:**

Our projections show that the WHO target to halt obesity by 2025 will most likely not be achieved. There is an urgent necessity for policy makers to implement effective prevent policies and other strategies in people who are at risk for developing overweight and/or obesity.

**Supplementary Information:**

The online version contains supplementary material available at 10.1186/s12889-022-13685-w.

## Background

In the European region, the prevalence of overweight increased from 48.0% in 1980 to 59.6% in 2015, and that of obesity from 14.5% in 1980 to 22.9% in 2015 [1]. Not only obesity is known as an important risk factor contributing to many negative health outcomes, such as cardiovascular disease, type 2 diabetes, colorectal, prostate and renal cancer, and many other non-communicable diseases [2–8], but also overweight is associated with these same negative health outcomes, albeit to a less severe extend [9].

Because of the large increase in cases in the past decade, overweight and obesity are now both considered as a global epidemic [10]. Belgium is no exception to this situation: obesity prevalence rose from 9.0% in 1978 to 15.0% in 1993 in a subsample of working, middle-aged men [11], and kept rising since. Consequently, a global obesity target to halt obesity at 2010 levels by 2025 was adopted during the World Health Assembly in 2013 [12], whereby consensus was reached to prevent a further growth in the number of men and women with obesity above the observed prevalence in 2010.

As obesity has been recognized as a major public health problem in Europe, research on its likely evolution in European countries is necessary. To this end, a variety of statistical models has been applied to the European context, such as linear extrapolations and wave functions. The diversity in methodologies employed differed substantially, and not all were able to yield realistic obesity estimates for the near future [13–22]. To better comprehend the complexity underlying the overweight and obesity epidemic, and obtain reliable and valid projections, it is important to consider the contributing risk factors. These factors may include socio-demographic factors such as age, sex, and education, but also lifestyle habits such as sedentary lifestyles and the consumption of high-calorie foods [1, 23–25]. For example, there is a known sex gradient in the impact of overweight and obesity, whereby the risk for developing asthma [26] and diabetes [27] is different between men and women. In addition, it is important to consider the so-called generation effect, i.e. the degree to which individuals are receptive to societal and social changes. These effects specifically feature the common exposures of people from the same birth cohort, such as common nutritional habits, smoking habits, and others [28–31]. Hence, the birth of age-period-cohort (APC) analysis, whereby the age effect reflects the association between age and weight status, the period effects reflects the evolution of weight over time, and the birth cohort effect reflects the degree to which an individual’s weight is receptive to societal and social changes.

An important issue in trend analysis is the limited availability of longitudinal data at regular time intervals [32]. Instead, epidemiologists often have to rely on repeated cross-sectional data to obtain long-term trends in overweight and obesity, whereby only basic information on age and sex is available for future populations based on population projections. Here is where the APC analysis excels as its estimated projections consider disentangled trends in age, period and cohort (i.e. generation) for overweight and obesity.

To date, only a limited number of studies have undertaken APC analyses to the prevalence rates of overweight and obesity, among which the number of European studies is scarce [10, 24, 33–35]. In addition, many of these studies did not consider the complex sampling process of the population microdata at hand, or have built further on the recent critiques regarding APC analysis [36, 37]. Lastly, the current pool of available studies mainly focused on estimating the APC effects, but the APC-analysis could be expanded by including other specific effects such as sex, and education. Therefore, the current study aims to (1) evaluate the age-period-cohort and other important effects based on the past and current data, and (2) estimate case projections using a APC modelling approach for a period of 10 years. To this end, a flexible Bayesian hierarchical APC model will be applied to population microdata available through the Belgian Health Interview Survey.

## Methods

### Data

Statistical analyses were performed using the datasets of the Belgian Health Interview Survey (BHIS), which was organized for the first time in 1997 by Sciensano, the Belgian Institute for Health, and contains a series of repeated cross-sectional sample surveys [38–40]. So far, six national health surveys have been carried out – in 1997, 2001, 2004, 2008, 2013 and 2018. The goal was to collect information on lifestyle and chronic diseases for approximately 10,000 individuals in each survey round. The survey applies a stratified multistage, clustered sampling method for each survey year. The combination of the large net sample size and the elaborate sampling methodology ensures that each sample is representative for the Belgian population, and that any trend found using the subsequent health surveys can be generalized to the larger Belgian population. More details on the sampling procedure have been published elsewhere (Demarest et al., 2013). The sole inclusion criteria to be eligible for the health interview study is a registration in the national register. For the current study, participants were only included if they had an age larger than or equal to 18. The survey was carried out in line with the Belgian privacy legislation and approved by the ethical committee of Ghent University.

In the current study, information on age, sex, education level, migration background, urbanization level, income level, and self-reported height and weight of participants was included from the different waves of BHIS [39].

The education level was used as a proxy indicator of the socio-economic status of the household and all its members. This indicator is based on the highest education level of the reference person or his/her partner and allocated to each member of the household. Possible values are “primary or no degree”, “secondary inferior”, “secondary superior”, and “superior education” following the ISCED-11 classification, whereby superior education includes all obtained degrees higher than secondary superior [41].

The income level is calculated based on the ‘total available income of the household’, for which an equivalent scale is applied [42]. This allows comparing incomes of different households taking their size and composition into account. The different members of the household receive a specific weight: 1.0 for the first adult member of the household, 0.5 for each additional adult (18+ years) and 0.3 for each child (< 18 years). The total available income of the household is divided by the sum of the weights of all the members of the household to calculate the equivalent income. The income levels (quintiles) include “< 750 euro”, “750–1000 euro”, “1000–1500 euro”, “1500–2500 euro”, and “> 2500 euro”, which are hereafter referred to as quantiles 1 to 5.

The level of urbanization was determined based on morphological and functional characteristics of the municipalities. Two morphological criteria are used to classify the municipalities: the population density and the area of habitation. Three criteria are used to describe the functional characteristics of the municipalities: the commercial function, the educative function and the employment rate. Based on these attributes, municipalities are labelled as “Big cities and dense agglomerations”, “Suburban”, “Urbanized municipalities”, or “Rural”.

The estimate for body mass index (BMI) was based on self-reported height and weight, and categorized as normal weight (0–25 kg/m^2^), overweight (≥ 25 kg/m^2^), and obese (≥ 30 kg/m^2^) [43].

Population projection data were retrieved from the Belgian Federal Planning Bureau (https://www.plan.be/publications), Belgium [44].

### Data analysis

To model the number of cases with overweight and obesity using age, period, and cohort effects, an age-period-cohort (APC) analysis was performed. We have built two separate models, one with overweight and one with obesity as dependent variable.

#### Independent variable

As explanatory variables, the models considered sex, education level, migration background, urbanization level, and income level as fixed independent categorical effects, and the categorical age, period and cohort effects as random effects. In the forecasting model, fixed effects that were not available as strata in the demography projections from the Belgian Federal Planning Bureau were excluded.

#### Dependent variable

The dependent variable was obesity and overweight, which were dichotomized based on the BMI of each individual.

#### Statistical model

One of the major issues in classical APC models is the linear dependency between the age effect, the period effect, and cohort effect. To tackle this linear dependency, the hierarchical age-period-cohort (HAPC) model has been introduced, which can include a mix of fixed and random effects [45–47]. Applying these HAPC models in a Bayesian framework allows for a direct interpretation of future trends in terms of credibility (e.g., how likely will overweight and obesity increase by at least 10%), whereas in the frequentist setting the projected uncertainty intervals cannot be interpreted as credibility. Hence, Bayesian HAPC models have been applied more frequent to forecast future trends in prevalence and incidence of cancer [48, 49], but to our knowledge, no study has applied the Bayesian APC model to forecast future trends in the prevalence of overweight and obesity.

Using the Bayesian framework, APC models [48] were fitted using the INLA package (Version 20.3.17) [50]. INLA stands for Integrated Nested Laplace Approximation, a novel approach that makes Bayesian inference faster compared to the computer-intensive Bayesian Markov chain Monte Carlo methods. More information on the INLA package can be found elsewhere (https://www.r-inla.org/). The number of overweight or obese individuals, y_ij_, in age group i and period j was modelled as a Binomial process with the mean equal to the product of the population at risk, N_ij_, and the estimated prevalence. The logit of the prevalence, η_ij_, was estimated as a linear combination of the age, period and cohort effects, respectively α_i_, β_j_ and γ_k_, where k = M(I − i) + j is the birth cohort, M is the number of periods per age group and I is the number of age groups.

$${\mathrm{y}}_{\mathrm{ij}}\sim \mathrm{Binomial}\left({\mathrm{N}}_{\mathrm{ij}},\mathrm{expit}\left({\upeta}_{\mathrm{ij}}\right)\right)$$

$${\upeta}_{\mathrm{i}\mathrm{j}}={\upalpha}_{\mathrm{i}}+{\upbeta}_{\mathrm{j}}+{\upgamma}_{\mathrm{k}}$$

Second-order random walk priors (RW2) were applied to the age, period and cohort effects. These RW2 effects are particularly well-suited to model unequal time intervals in the APC effects [51]. Log-gamma priors were applied to the precision parameters with scale and shape parameters of 1 and 0.00005 for each of the age, period and cohort effects. Modelling priors were based on the methodology from Cameron & Baade (2019) and Riebler & Held (2010). Model selections for factors and priors was based on information criteria: DIC, Deviance information criterion, and WAIC, Watanabe–Akaike information criterion. Projected temporal trends were described as the median and 95% credibility intervals (CrI) around the median were constructed based on the 2.5 and 97.5% quantile of the posterior distribution.

In addition, based on the marginal posterior distribution of the modelled count, we calculated the probability, P(y_t + x_ > y_t_ + m × y_t_), that a projected rate in year t + x is greater than the modelled value in the final year of observed data (t = 2018), by some margin m (expressed as percentage increase, %). All statistical analyses were performed in R 4.1.0 [52].

### Model validation

Model fit and predictive accuracy were assessed by fitting the model to the repeated cross-sectional sample surveys [38]. The explained variance (R^2^) and root mean squared error (RMSE) between the observed and projected prevalence counts were calculated for different priors and models. More details on the model validation are provided in Supplementary material A.

## Results

### Population

**Fig. 1:**
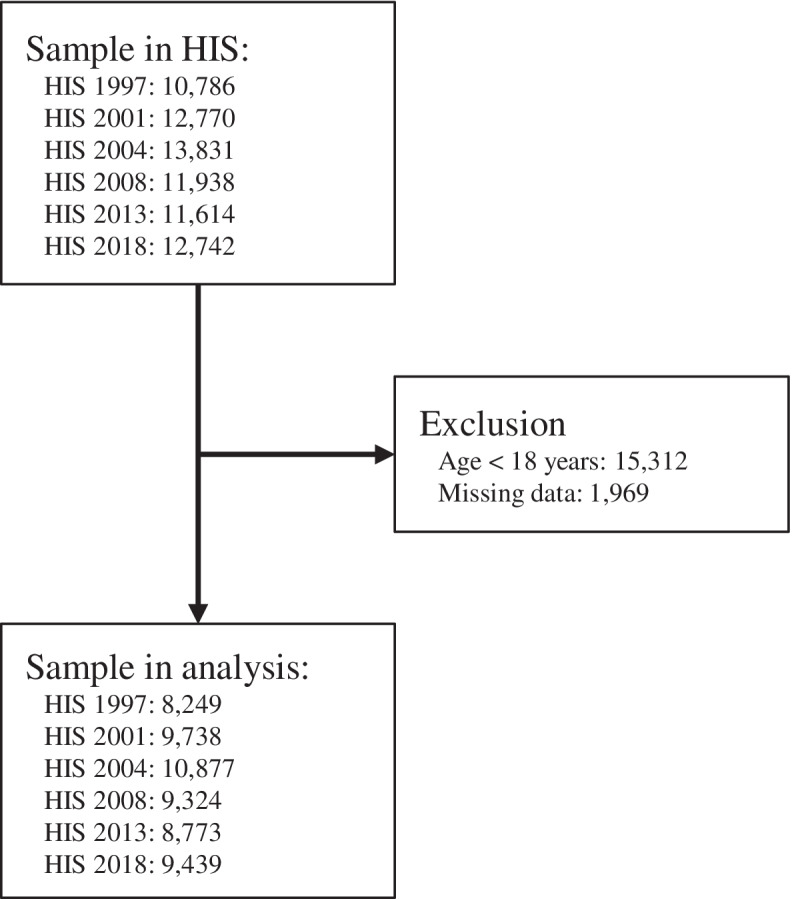
Participants flowchart according to in- and exclusion criteria

In total, the sample included 73,681 participants across all surveys. More information on the final sample that was selected for the analysis sample is depicted in the flowchart (Fig. 1). The raw socio-demographic, and health-related characteristics of each cohort are listed in Table 1. The average age across the different cross-sectional cohorts ranged from 44 to 51 years. The majority of participants were females with proportions ranging from 52 to 54%. Over time, the proportion of participants with a higher education has increased from 29% in 1997 to 41% in 2018. The level of urbanization has remained stable over time with the majority of participants living in big cities (47%), and a minority living in a suburban (13%) or rural area (15%) in 2018. The income distribution shows an increase of incomes in the higher quintiles (Quintile 4 and 5) and a decrease in the lower quintiles (Quintile 1 and 2). The number of non-EU immigrants has doubled from 5.2% in 1997 to 11.0% in 2018.

**Table 1:** Socio-demographic characteristics of the included participants by period

Characteristic	1997, ***N*** = 8471^a^	2001, ***N*** = 9949^a^	2004, ***N*** = 11297^a^	2008, ***N*** = 9637^a^	2013, ***N*** = 9051^a^	2018, ***N*** = 9964^a^
Age	44 (32, 61)	46 (33, 62)	51 (35, 69)	51 (33, 70)	48 (34, 63)	50 (35, 64)
Sex						
Man	4103 (48%)	4820 (48%)	5202 (46%)	4383 (46%)	4385 (47%)	4765 (48%)
Woman	4368 (52%)	5129 (52%)	6095 (54%)	5246 (54%)	4762 (53%)	5199 (52%)
Obesity (≥ 30 kg/m^2^)						
No	7585 (90%)	8781 (88%)	10,011 (89%)	8466 (88%)	7846 (87%)	8508 (85%)
Yes	886 (10%)	1168 (12%)	1286 (11%)	1171 (12%)	1205 (13%)	1456 (15%)
Overweight (≥ 25 kg/m^2^)						
No	5098 (60%)	5735 (58%)	6746 (60%)	5655 (59%)	4912 (54%)	5300 (53%)
Yes	3373 (40%)	4214 (42%)	4551 (40%)	3982 (41%)	4139 (46%)	4664 (47%)
Diploma						
No diploma or primary education	1663 (22%)	1915 (21%)	2239 (22%)	1666 (20%)	1229 (15%)	905 (10%)
Lower secondary	1653 (21%)	1913 (21%)	2023 (20%)	1650 (19%)	1350 (17%)	1471 (17%)
Higher secondary	2155 (28%)	2546 (28%)	2814 (28%)	2528 (30%)	2607 (32%)	2775 (32%)
Higher	2248 (29%)	2590 (29%)	2975 (30%)	2684 (31%)	2919 (36%)	3526 (41%)
Urbanisation						
Big city	4368 (52%)	4644 (47%)	5025 (44%)	4785 (50%)	4283 (47%)	4649 (47%)
Suburban	1038 (12%)	1527 (15%)	1528 (14%)	1268 (13%)	1221 (13%)	1286 (13%)
Urbanized municipality	1924 (23%)	2213 (22%)	2649 (23%)	1878 (19%)	1817 (20%)	2547 (26%)
Rural	1141 (13%)	1565 (16%)	2095 (19%)	1706 (18%)	1730 (19%)	1482 (15%)
Income						
Quintile 1	1969 (24%)	1968 (23%)	2184 (23%)	1727 (23%)	1677 (22%)	1133 (14%)
Quintile 2	1584 (20%)	1585 (18%)	1829 (19%)	1435 (19%)	1321 (17%)	1311 (16%)
Quintile 3	1623 (20%)	1600 (19%)	1858 (20%)	1537 (20%)	1542 (20%)	1606 (19%)
Quintile 4	1465 (18%)	1668 (19%)	1711 (18%)	1153 (15%)	1512 (19%)	2061 (25%)
Quintile 5	1412 (18%)	1754 (20%)	1901 (20%)	1806 (24%)	1724 (22%)	2190 (26%)
Nationality						
Belgian	7186 (87%)	8787 (90%)	9718 (90%)	8142 (88%)	7481 (85%)	7920 (80%)
EU	633 (7.7%)	586 (6.0%)	698 (6.4%)	779 (8.4%)	809 (9.2%)	975 (9.8%)
Non-EU	428 (5.2%)	347 (3.6%)	430 (4.0%)	380 (4.1%)	476 (5.4%)	1065 (11%)

*N* number of participants

^a^Median (IQR); n (%);^*^Total may not be equal to N due to missing or undefined category

### Past and present trends in overweight and obesity

The rate of overweight in the population adjusted for the survey design increased by 8.9% from 45,348 [45,192; 45,504] per 100,000 in 1997 to 49,412 [49,257; 49,567] per 100,000 in 2018, while the rate of obesity in the population increased by 25.9% from 12,978 [12,892; 13,066] in 1997 to 16,339 [16,238; 16,440] in 2018. Summary measures of model fit in terms of DIC and WAIC for the overweight and obesity model are presented in Table 2. DIC and WAIC were the lowest for the model including age, period, and cohort effects together with all fixed effects (sex, urbanisation level, education level, income and nationality). Sex (Woman vs Man) was identified as a risk factor for overweight (OR = 0.55; 95% CrI = [0.55; 0.55]), but was a negligible risk factor for obesity (OR = 0.97; 95% CrI = [0.96; 0.97]). Superior education (OR = 0.67; 95% CrI = [0.66; 0.67]) was associated with decreased odds for overweight compared to no education or primary degree, whereas both superior education (OR = 0.49; 95% CrI = [0.49; 0.49]) and higher secondary education (0.74; 95% CrI = [0.74; 0.75]) were associated with decreased odds for overweight compared to the group with no education or primary degree. Middle incomes (Quantile 3) showed a higher risk of obesity and overweight compared to the lowest incomes (Quantile 1), but the highest incomes (Quantile 5) showed a reduced risk with an estimated OR of 0.92 [0.92; 0.92] for overweight and 0.83 [0.83; 0.84] for obesity. More information on the estimated fixed effects for obesity and overweight can be found in Table 3.

**Table 2:** Model selection for past trends of overweight and obesity

Model	DIC	WAIC
Overweight		
APC	59,870,210	43,824,652
AP	60,028,035	43,825,596
AC	60,490,979	44,267,784
P	63,242,295	47,049,883
APC + F	**58,456,210**	**42,435,966**
Obesity		
APC	33,646,425	19,654,269
AP	33,770,297	19,607,961
AC^a^	NA	NA
P^a^	34,992,302	19,899,193
APC + F	**33,142,627**	**19,542,554**

^a^Only converted when prior values were fixed

*Abbreviations*: *APC* Age-Period-Cohort, *AP* Age-Period, *AC* Age-Cohort, *P* Period, *APC + F* Age-Period-Cohort and covariates, *DIC* Deviance information criterion, *WAIC* Watanabe–Akaike information criterion

**Table 3:** Multivariable analysis of risk factors for overweight and obesity based on Bayesian age-period-cohort model

	Overweight			Obesity		
	Odds ratio	95% Credible interval		Odds ratio	95% Credible interval	
		lower	upper		lower	upper
**Fixed effects**						
Intercept	0.783	0.747	0.819	0.124	0.119	0.129
Sex						
Man (Ref.)						
Woman	0.545	0.545	0.546	0.966	0.964	0.968
Urbanisation level						
Urban (Ref.)						
Suburban	1.023	1.021	1.025	0.992	0.989	0.994
Urbanized municipality	1.049	1.048	1.051	1.005	1.003	1.007
Rural	1.095	1.092	1.097	1.038	1.035	1.041
Education level						
No diploma /primary education (Ref.)						
Lower secondary	0.997	0.995	0.999	0.934	0.932	0.937
Higher secondary	0.918	0.916	0.920	0.744	0.742	0.746
Superior education	0.665	0.664	0.666	0.491	0.489	0.492
Income level						
Quintile 1 (Ref.)						
Quintile 2	1.166	1.164	1.168	1.123	1.120	1.126
Quintile 3	1.092	1.090	1.094	1.138	1.135	1.141
Quintile 4	1.087	1.085	1.089	0.951	0.948	0.954
Quintile 5	0.922	0.920	0.923	0.834	0.832	0.837
Nationality						
Belgian (Ref.)						
EU	1.064	1.061	1.067	1.023	1.019	1.027
Non-EU	1.142	1.138	1.146	0.969	0.964	0.974
**Random effects** (with RW2-specification)						
Age						
18–25 (ref.)						
26–30	2.175	2.172	2.179	2.270	2.264	2.276
31–35	2.383	2.376	2.390	2.329	2.319	2.339
36–40	2.825	2.814	2.837	3.109	3.090	3.129
41–45	3.387	3.369	3.405	3.851	3.820	3.883
46–50	3.505	3.483	3.528	3.158	3.128	3.188
51–55	3.691	3.664	3.717	3.695	3.655	3.736
56–60	4.055	4.025	4.086	3.769	3.726	3.813
61–65	3.452	3.426	3.478	2.846	2.814	2.879
66–70	3.494	3.470	3.518	2.627	2.599	2.656
71–75	2.868	2.850	2.885	1.996	1.977	2.014
76–80	2.530	2.518	2.543	1.580	1.569	1.593
81–85	1.642	1.636	1.648	0.948	0.943	0.953
86–90	1.246	1.244	1.248	0.585	0.584	0.586
91–95	0.818	0.816	0.820	0.442	0.439	0.444
95+	0.410	0.401	0.419	0.492	0.477	0.506
Period						
1997 (Ref.)						
2001	1.107	1.106	1.108	1.147	1.145	1.148
2004	1.107	1.106	1.109	1.249	1.246	1.252
2008	1.351	1.349	1.354	1.560	1.556	1.564
2013	1.576	1.574	1.577	1.779	1.776	1.781
2018	1.775	1.774	1.775	2.378	2.377	2.379
Cohort (Birth year)						
(1895, 1900] (Ref.)						
(1900, 1905]	5.390	3.000	18.549	2.822	1.891	5.880
(1905, 1910]	29.512	15.812	105.989	8.220	5.126	18.627
(1910, 1915]	19.936	10.650	71.827	8.039	4.949	18.475
(1915, 1920]	30.645	16.360	110.494	10.393	6.379	23.958
(1920, 1925]	28.771	15.353	103.782	10.016	6.140	23.121
(1925, 1930]	33.093	17.656	119.400	9.760	5.980	22.543
(1930, 1935]	29.198	15.575	105.364	7.680	4.704	17.747
(1935, 1940]	28.567	15.237	103.098	7.511	4.599	17.360
(1940, 1945]	25.543	13.623	92.190	7.566	4.632	17.493
(1945, 1950]	24.069	12.836	86.872	6.350	3.887	14.682
(1950, 1955]	20.019	10.677	72.253	5.666	3.469	13.100
(1955, 1960]	17.680	9.430	63.807	4.034	2.470	9.325
(1960, 1965]	16.753	8.936	60.458	4.187	2.564	9.677
(1965, 1970]	17.136	9.142	61.829	4.043	2.477	9.340
(1970, 1975]	15.777	8.418	56.915	3.908	2.395	9.022
(1975, 1980]	15.049	8.032	54.278	3.510	2.153	8.100
(1980, 1985]	14.115	7.535	50.894	3.759	2.306	8.667
(1985, 1990]	13.220	7.059	47.652	3.560	2.186	8.202
(1990, 1995]	12.013	6.417	43.284	2.592	1.594	5.964
(1995, 2000]	13.514	7.222	48.669	3.116	1.918	7.161

*Ref.* reference

**Fig. 2:**
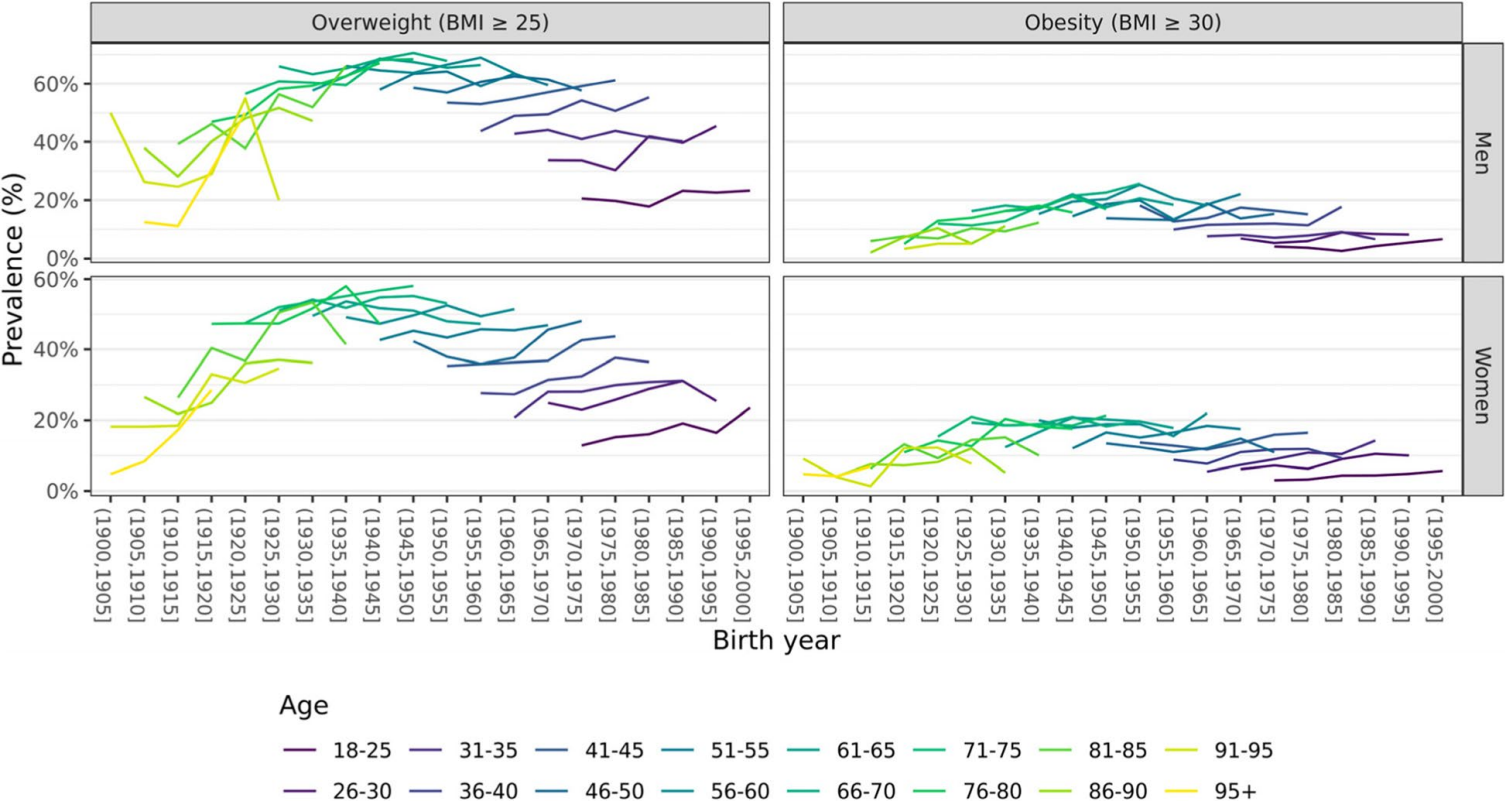
Observed prevalence rates for overweight and obesity. Plotted versus birth year by age group

**Fig. 3:**
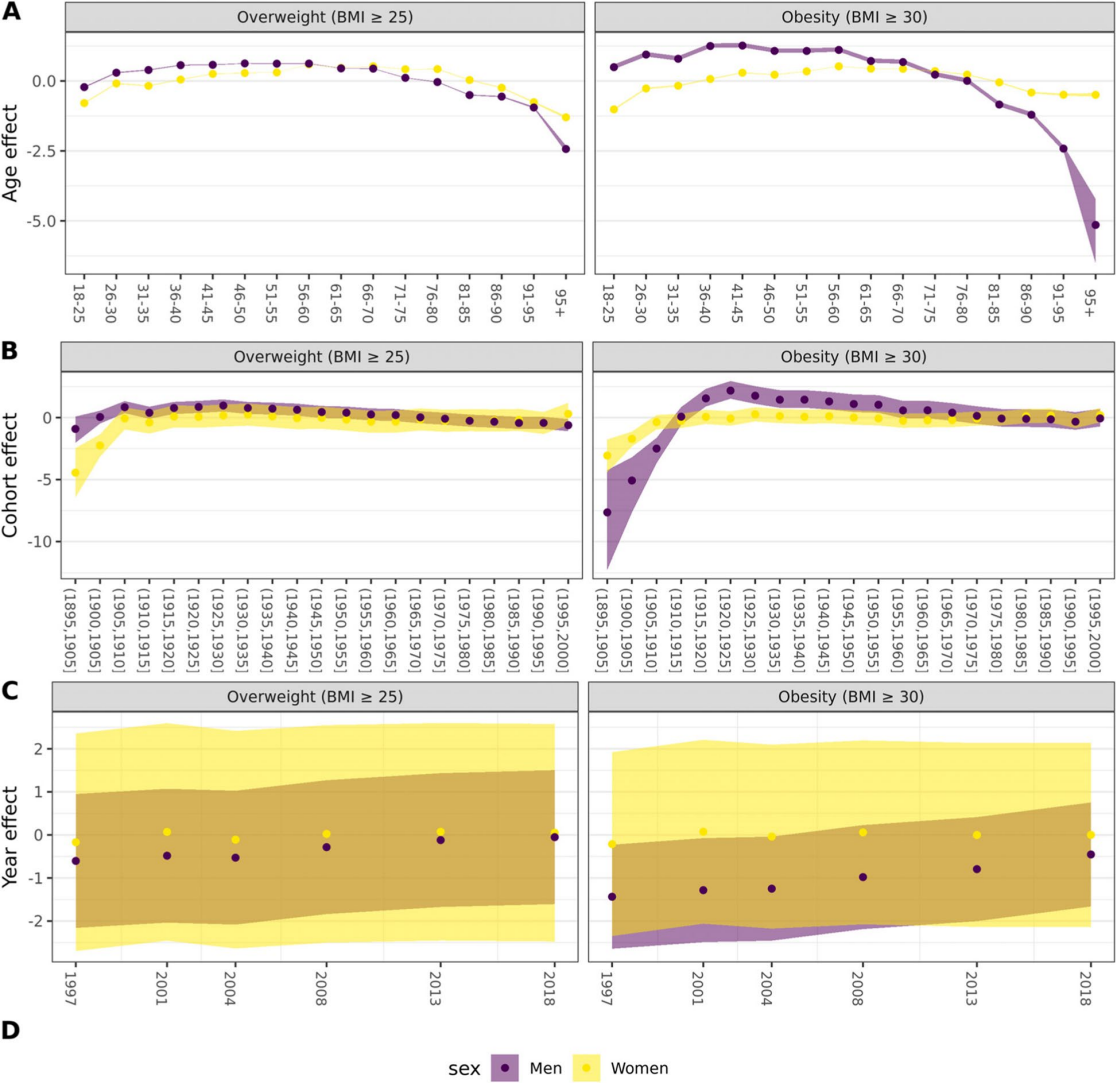
Modelled effects for age, period, and cohort. Plotted by sex. The dots represent the estimated value of the coefficients for each of the hierarchical effects in the Bayesian HAPC model. The areas reflect the 95% credibility intervals expressed as quantile in men and women

Figures 2 and 3 depicts the disentangled age, cohort, and period effects by sex for the occurrence of overweight and obesity in the Belgian population. The prevalence of overweight and obesity is the highest for the middle-aged groups in both men and women. In addition, the prevalence of overweight and obesity has increased over time in men and women. In men, the observed increase was stronger compared to the increase in women. Lastly, there was a clear increase in the prevalence of obesity and overweight among the oldest generations. However, since the generations born from 1915 and onwards the cohort effect has remained relatively stable.

### Future trends in overweight and obesity

**Fig. 4:**
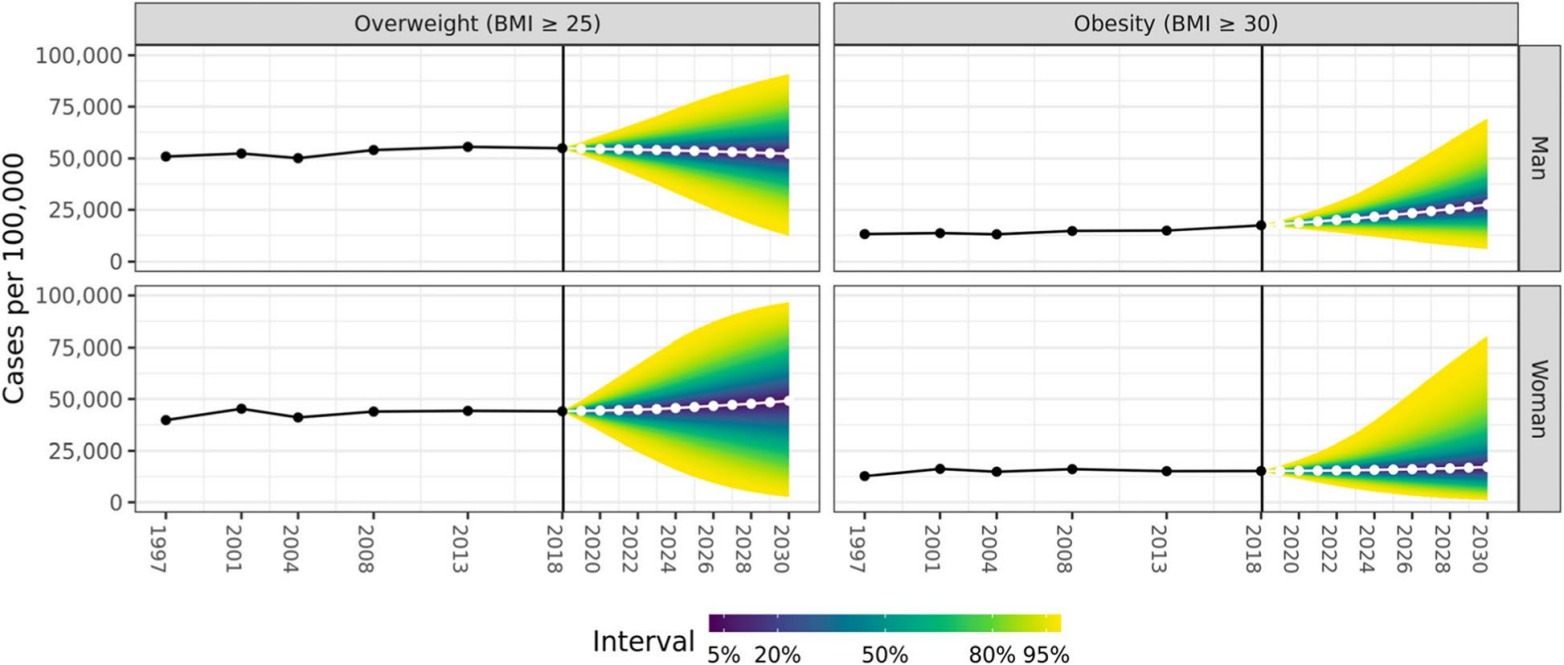
Predicted prevalence rates for overweight and obesity. Plotted by year. The modelled observed data is depicted in black. Projections are depicted from the vertical line onwards for the period 2019–2030 in white, with quantiles from the estimated marginal posterior distribution of the projected prevalence. The colours reflect the credibility intervals expressed as quantile, coloured consecutively from dark blue to yellow

Modelled and projected rates with their 95% credibility interval (CrI) for overweight and obesity are given by sex in Fig. 4. The explained variance in prevalence expressed as the coefficient of determination (R^2^) by the overweight model equalled 80.6 and 76.0%, and the explained variance in prevalence by the obesity model equalled 76.7 and 58.3% for men and women, respectively.

Temporal trends in rates for overweight in men showed a steady state to a slight decrease from 54,764 [54,764; 55,125] per 100,000 in 2018 towards a projected 53,616 [36,291; 70,866] cases per 100,000 in 2025, and 52,273 [21,216; 83,249] cases per 100,000 in 2030. In women, the projection showed a potential strong increase in cases from 44,070 [43,884; 44,255] per 100,000 in 2018 towards a projected 46,147 [19,666; 74,114] per 100,000 in 2025, and 49,147 [7275; 90,899] per 100,000 in 2030. In contrast, a high increase was observed for obesity in men from 17,453 [17,322; 17,585] cases per 100,000 in 2018 towards 22,497 [13,622; 35,616] cases per 100,000 in 2025, and 27,566 [9938; 57,358] cases per 100,000 in 2030. In women, the rate of increase was almost as high compared to men with cases increasing from 15,246 [15,125; 15,369] cases per 100,000 in 2018 towards 15,954 [6354; 35,020] cases per 100,000 in 2025, and 17,186 [2552; 61,803] cases per 100,000 in 2030.

Probabilities for exceeding a certain threshold m (> 0%, > 5%, > 10%, > 25%, > 50%, and > 100%) based on the marginal posterior distribution of the modelled counts are listed in Table 4. In men, there is a 43.3% probability for an increase in the rate of overweight by the year 2025 and 2030. The probability for an increase in the number of men with obesity by the year 2025 and 2030 are much higher in comparison, with estimated probabilities of 86.7% and 84.1%, respectively. In women, there is a 56.0% and 57.4% probability for an increase in the rate of overweight by the year 2025 and 2030, respectively. The probability for an increase in the number of women with obesity by the year 2025 and 2030 are much higher in comparison, with estimated probabilities of 54.6% and 56.0%, respectively.

**Table 4:** Probability of increase in rate by > 0, > 5, > 10, > 25, > 50 and > 100% based on the marginal posterior distribution of future projections based on the Bayesian APC-model

	Overweight				Obesity			
	Men		Women		Men		Women	
	2025	2030	2025	2030	2025	2030	2025	2030
Increase > 0%	43.3%	43.3%	56.0%	57.4%	86.7%	84.1%	54.6%	56.0%
Increase > 5%	30.4%	36.6%	49.6%	54.2%	81.9%	81.6%	49.6%	53.5%
Increase > 10%	19.9%	30.4%	43.2%	50.9%	76.2%	78.9%	44.8%	51.2%
Increase > 25%	4.0%	15.3%	25.7%	41.0%	55.6%	70.1%	32.0%	44.5%
Increase > 50%	0.1%	2.8%	7.6%	25.2%	24.4%	54.7%	17.3%	35.1%
Increase > 100%	< 0.1%	< 0.1%	0.1%	3.8%	2.9%	28.3%	4.9%	21.4%

**Fig. 5:**
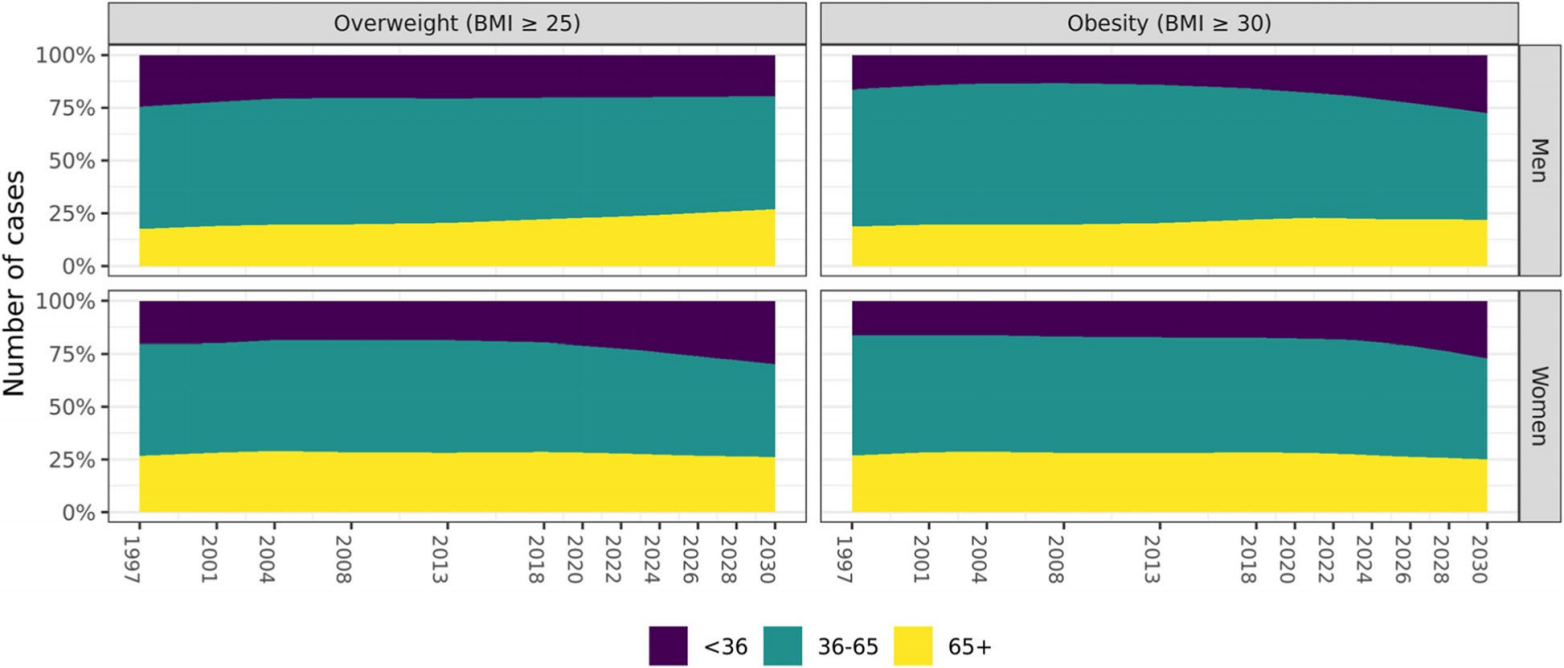
Predicted prevalence rates for overweight and obesity plotted by age group and year

As depicted in Fig. 5, the age structure of men and women with overweight and obesity has changed over time. In 2018, 57.7% and 61.8% of men, respectively with overweight and obesity, had an age between 36 and 65 years. Similarly, 51.5% and 53.9% of men, respectively with overweight and obesity, had an age between 36 and 65 years. The current projections estimate that these proportions will drop in 2030 to 53.6% and 50.4% in men, and 43.8% and 47.8% in women, whereby the proportion of men and women with obesity will mainly increase in the youngest (< 36 years) age category.

## Discussion

Our analyses based on nationally representative data collected over six large scale health interview surveys, covering a period spanning over 20 years from 1997 to 2018, showed a disturbing picture of the future overweight and obesity epidemic in Belgium, assuming similar future increase rates as observed in the past. In men, an increase in overweight is less likely, whereby the probability of an increase by 2030 equals 43.3%. In contrast, an increase in obesity by 2030 is very likely with a probability of 84.1%. In women, an increase in overweight and obesity will likely be reached by 2030 with a respective probability of 57.4% and 56.0%.

### Past and current trends in overweight and obesity

The age, period and cohort effects are similar to those reported in previous reports [1, 35], whereby the number of men and women with overweight and obesity has increased over time among all generations. The current trends indicate that overweight and obesity do not occur among specific generations, but rather affect all generations with an increasing trend over time among all generations. In addition, higher prevalence rates of overweight and obesity were demonstrated among middle-aged women and men. More specifically, among Belgians, a middle-age man, born before 1970 with no higher education and a middle income has the highest risk for being overweight and obesity at present. Among these risk factors, the highest were sex and education. It is well-known that sex plays an important role in the metabolic and genetic predisposition of overweight and obesity, whereby overweight and obesity do not only occur more frequent among men, but also yield an increased risk for developing overweight-related disorders among men [53, 54]. Socio-economic factors also play an important role in the occurrence of overweight, whereby healthier behaviour could be driven by a higher health-literacy, which in its turn relates to higher education levels [55].

### Projections in overweight and obesity

Our projections indicate that Belgium is unlikely to meet the global obesity target to halt obesity “at 2010 levels” by 2025, which was adopted during the World Health Assembly in 2013 [12]. The current projected prevalence rates for overweight and obesity will likely be higher in both sexes compared to their respective 2010 prevalence estimates. In another study, it was estimated that the global rate of overweight in established economic markets would rise to 36.3% by 2030 following population projections and a steady prevalence rate of 2005. The same study estimated an increase to 30.0% when modelling the prevalence based on past data. Likewise, the obesity prevalence was estimated at 22.1% and 36.2% [21]. These estimates are lower compared to ours, however, the observed prevalence in 2018 already exceeded the projection for overweight by 10.6%, which indicates that these previous estimates were rather too conservative. Similar to our results, most study reports agree that the growth in cases with obesity is faster compared to the growth in cases with overweight [13–22]. The increase in overweight and obesity likely results from a complex interaction between changes in the food environment, physical activity, socioeconomic, environmental, and genetic factors [1]. For example, the number of low quality away from home food consumptions, a known environmental risk factor for overweight and obesity [56], has grown over the years. Assuming trends in behaviour are likely to evolve similarly to the past, the number of cases with overweight and/or obesity are very likely to increase further. Efforts have been made to halt the rise of overweight, but prevention is a complex issue and requires collective efforts from the governments, the scientific and the medical communities, the industry, and various social organizations towards the changing of dietary and lifestyle habits.

### Policy implications

We argue, in accordance with the current evidence, that a multi-faceted approach will be necessary to halt a further increase in cases. This approach should include policy guidelines and legislation that focuses on prevention, but also include treatments that are considered effective for people with overweight and obesity. The main pillar to reach a halt in cases is the prevention of overweight and obesity. Prevention should already start in childhood and early adolescence by implementing health promotion in schools including offering healthy snacks and meals, and promoting sufficient levels of physical activity [57]. Further prevention can be achieved by implementing the prioritized food environment policies [58]. If prevention fails, healthcare workers can rely on effective treatment strategies, which mainly focus on lifestyle and behavioural changes in nutrition and physical activity, or – in the worst case – pharmacotherapy or bariatric Surgery [59–61]. Next, it is important to consider the socio-demographic gradient in overweight and obesity. It is known that some groups are more prone to develop overweight or obesity. Therefore, it is important implement policies that target this socio-demographic gradient in cases [62].

Without taking action, the rise in cases will likely follow the projected trajectory, whereby the rates of disease burden and associated healthcare cost of non-communicable diseases will also rise [63, 64]. The fact that environmental and behavioural forces fuelling the obesity epidemic are unlikely to be modified overnight, and even effective prevention programs may take years to show a significant impact [13], further emphasized the importance of prioritizing overweight and obesity on the healthcare agenda.

Lastly, the current forecasted rate of increase might have been exacerbated by the COVID-19 pandemic, as government introduced a myriad of measures such as cancellation of small gatherings, quarantine, lockdowns, and individual movement restrictions to combat the spread of the coronavirus [65]. Consequently, decreases in physical activity and increases in sedentary behaviours during lockdowns have been reported by different studies [66, 67], resulting in a potential increase of overweight and obesity in the current generations [68].

### Strengths and limitations

APC models account for trends in risk factors, without requiring measurements of exposures. Projected counts are hence susceptible to unforeseeable changes, which might result in an over- or underestimate the actual future prevalence. The key assumption in the APC models is, as with any projection model, a continuation of the observed historical trends. However, evaluating the validity of that assumption is impossible.

Despite the wide uncertainty in projections beyond 5 or 10 years, governments require longer term projections for effective planning and policy development. The increased uncertainty provides a more realistic picture of the accuracy of projections, particularly when unforeseen interventions and changing circumstances (e.g. COVID-19) that could take place are considered. Since the cohort is a linear combination of age and period, the effects are not identifiable and cannot provide statistical evidence of change in prevalence [69]. Nevertheless, the models provide information on the prevalence and distribution across the population. The current study includes a large population of individual data over a time-span of 10 years. These data were modelled using a well-described modelling strategy including the incorporation of weights according to the sampling procedure, which results in realistic projections of future scenarios.

BMI has been defined according to the WHO definition, and based on self-reported estimates for weight and height. Consequently, the actual number of participants with obesity and overweight might have been higher as BMI is on average underestimated by 0.97 kg/m^2^ [70].

In a next step, future research could focus on integrating policy-decisions based on a scenario-based modelling approach. For example, including information on the level of physical activity or nutritional habits, and associated policy interventions could be expressed in terms of the changes in projected rate.

## Conclusions

In conclusion, an alarming increase in the prevalence of overweight and obesity is predicted with a very likely increase in the prevalence of overweight and obesity among Belgian adults within the next 10 years. There is an urgent need to implement food environment policies, support preventive strategies, and support effective treatments to halt the increase in cases with overweight and obesity.

## Supplementary Information

**Additional file 1.**

## Data Availability

The data that support the findings of this study are available from the Health Interview at Sciensano but restrictions apply to the availability of these data, which were used under license for the current study, and so are not publicly available. Data are however available from the authors upon reasonable request and with permission of Health Interview team at Sciensano.
