# Supplementary material for: Past, present, and future trends of overweight and obesity in Belgium using Bayesian age-period-cohort models
Source: BMC Public Health. 2022 Jul 7;22:1309. doi: 10.1186/s12889-022-13685-w (PMC9263047; doi:10.1186/s12889-022-13685-w)
Supplement: Supplementary file 1 — Additional file 1. [file 12889_2022_13685_MOESM1_ESM.docx]

**SUPPLEMENTARY MATERIAL A**

The Bayesian APC model is based on the methodology described by Riebler and Held [33]. The prevalence of overweight and obesity was modelled using a Bayesian binary logistic regression model including a logit-link function. The binary logistic regression model structure was formulated as ${logit(P(y=1))}_{ijkl}=\mu+a_{j}+p_{k}+c_{l}(+\sum_{c} \beta_{c}x_{i})$, where $\mu$ is the intercept, $a_{j}$ the $j$th age-effect, $p_{k}$ the $k$th period-effect and $c_{l}$ the $l$th cohort-effect. The age, period and cohort effects were estimated using the random walk of second order (RW2).

Model priors (hyperparameters) were estimated based on a general linear mixed effects model (GLMER) by extracting the variance covariance for each of the random terms (age, period and cohort). Afterwards, log transformation on the inverse of the variance-covariance was calculated and applied as prior in the Bayesian INLA model ($initial= log(1/{vcov}$)).

The R code used for estimating the initial values of the RW2-models is:

fit_in1.mer0 <-

lmer(overweight ~ (1 | age_category) + (1 | year) + (1 | synthetic_cohort).

data = data)

vc0 <- as.data.frame(VarCorr(fit_in1.mer0))

vc0$init <- log(1 / vc0$vcov)

The R-code used for modelling the data based on a Bayesian INLA model:

proj_overweight <- inla(

as.numeric(as.character(overweight)) ~

f(age_category, model = "rw2",

hyper = list(prec = list(

initial = vc0$init[[2]],

scale.model = F,

param = c(1, 0.00005),

prior = "loggamma"

))) +

f(year, model = "rw2",

hyper = list(prec = list(

initial = vc0$init[[3]],

scale.model = F,

param = c(1, 0.00005),

prior = "loggamma"

))) +

f(synthetic_cohort, model = "rw2",

hyper = list(prec = list(

initial = vc0$init[[1]],

scale.model = F,

param = c(1, 0.00005),

prior = "loggamma"

))),

family = "binomial",

data = df_proj,

weights = df_proj$individual_weight,

quantile = seq(from = 0.001, to = 0.999, by = 0.001),

verbose = T,

control.compute = list(

dic = T,

waic = T,

cpo = T,

hyperpar = T,

mlik = T

),

control.predictor = list(compute = TRUE, link = 1)

)

***Model validation***

To calibrate and validate the Bayesian APC-model. different prior values were evaluated:

- Variance-Covariance value from GLMER.
- Fixed prior values at log(0.00001).

The Variance-Covariance values from the frequentist GLMER model showed the best fit on the observed data as depicted in ***figure 1***. The average squared error between the observed data and the predicted values (RMSE) for overweight and obesity equals 0.003 and 0.001 respectively. The coefficient of determination equals 87.7% for overweight and 73.3% for obesity.

*
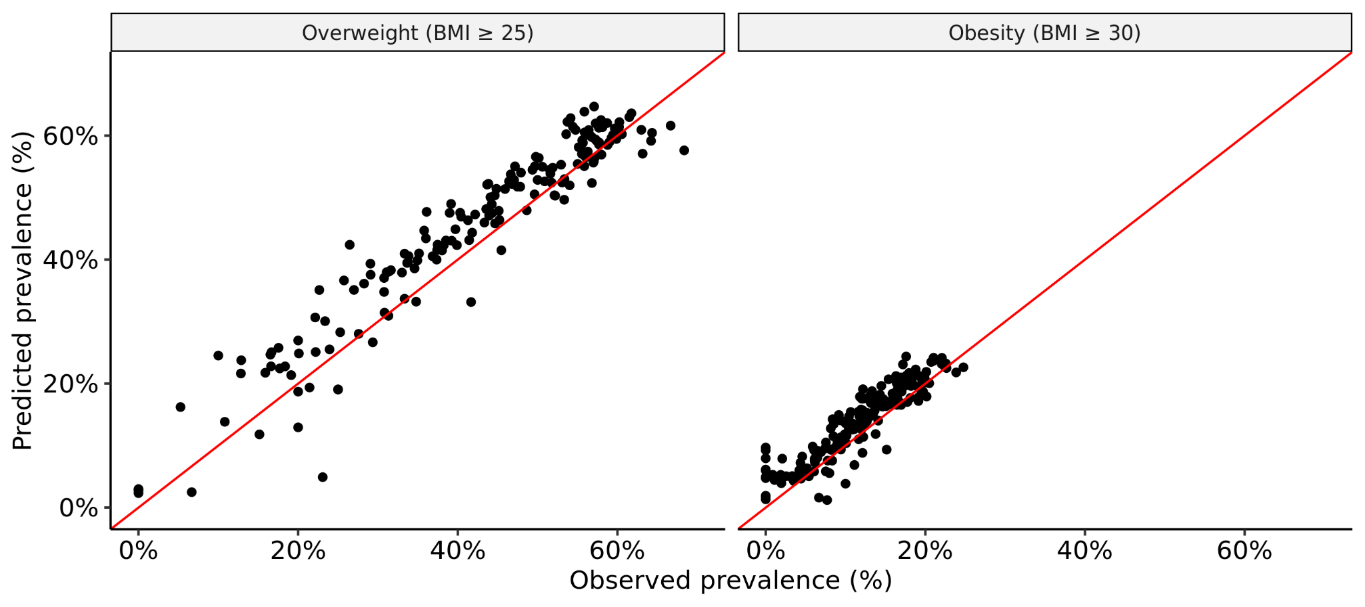
*
